# Prediction of Cardiovascular Events by Using Non-Vascular Findings on Routine Chest CT

**DOI:** 10.1371/journal.pone.0026036

**Published:** 2011-10-12

**Authors:** Pim A. de Jong, Martijn J. A. Gondrie, Constantinus F. M. Buckens, Peter C. Jacobs, Willem P. T. h. M. Mali, Yolanda van der Graaf, the PROVIDI study group

**Affiliations:** 1 Department of Radiology, University Medical Center Utrecht, Utrecht, The Netherlands; 2 Julius Center for Health Sciences and Primary Care, University Medical Center Utrecht, Utrecht, The Netherlands; University Medical Center Rotterdam, The Netherlands

## Abstract

**Background:**

Routine computed tomography (CT) examinations contain an abundance of findings unrelated to the diagnostic question. Those with prognostic significance may contribute to early detection and treatment of disease, irrelevant findings can be ignored. We aimed to assess the association between unrequested chest CT findings in lungs, mediastinum and pleura and future cardiovascular events.

**Methods:**

Multi-center case-cohort study in 5 tertiary and 3 secondary care hospitals involving 10410 subjects who underwent routine chest CT for non-cardiovascular reasons. 493 cardiovascular hospitalizations or deaths were recorded during an average follow-up time of 17.8 months. 1191 patients were randomly sampled to serve as a control subcohort. Hazard ratios and annualized event rates were calculated.

**Results:**

Abnormalities in the lung (26–44%), pleura (14–15%) and mediastinum (20%) were common. Hazard ratios after adjustment for age and sex were for airway wall thickening 2.26 (1.59–3.22), ground glass opacities 2.50 (1.72–3.62), consolidations 1.97 (1.12–3.47), pleural effusions 2.77 (1.81–4.25) and lymph-nodes 2.04 (1.40–2.96). Corresponding annual event rates were 5.5%, 6.0%, 3.8%, 10.2% and 4.4%.

**Conclusions:**

We have identified several common chest CT findings that are predictive for future risk of cardiovascular events and found that other findings have little utility for this. The added value of the non-vascular predictors to established vascular calcifications on CT remains to be determined.

## Introduction

The use of diagnostic imaging is increasing rapidly: in the United States of America approximately 62 million computed tomography (CT) examinations [1] are obtained annually in a population of some 300 million. Chest CT images exhibit an abundance of findings unrelated to the clinical problem which challenge physicians as they often lead to further testing, patient anxiety and unnecessary procedures for findings without relevance. On the other hand unrequested findings also provide an opportunity for early disease detection and prevention of symptoms and mortality [2], [3]. Therefore, reliable, reproducible and meaningful prognostic models need to be developed for treatable diseases and the impact of such models on patient outcome needs to be evaluated [4].

We hypothesized that certain findings in the lung, pleura and mediastinum on routine chest CT examinations might contain prognostic information for cardiovascular events. The hypothesis is based on the fact that airway obstruction is consistently found to be independently associated with cardiovascular mortality [5] and lung diseases are now coming to be seen as a risk factor for cardiovascular mortality [6], [7]. Several mechanisms including ongoing pulmonary inflammation [5], [8], repair mechanisms [9], misbalances between proteases and anti-proteases [8] and auto-immune phenomena [10] that damage the lung also appear to adversely affect the cardiovascular system. The advances and increased use of chest CT make the pulmonary manifestations of these processes increasingly accessible by direct in vivo visualization of the morphological signs [11] of obstructive pulmonary disease [10], [12]–[16] and chest inflammation [10], [12]–[18].

We believe that for an important subgroup of patients who undergo chest CT the cardiovascular risk is unknown and therefore that a cardiovascular risk calculation based on unrequested chest CT findings may reveal hidden cardiovascular disease. Cardiovascular diseases, despite their high prevalence [19] and high awareness of risk reduction measures, often manifest suddenly and unexpectedly in a large portion of patients with no prior cardiovascular disease history [20]. Identification of at-risk patients using unrequested findings on otherwise routine chest CT leading to timely diagnosis and concomitant treatment may be relevant as early treatment has potential to reduce risk of future cardiovascular events [21], [22].

The aim of this study was to assess the association between unrequested chest CT findings in lungs, mediastinum and pleura and future cardiovascular events in a cohort of routine diagnostic chest CTs performed on adults in the Netherlands.

## Methods

### Ethics statement

This study was approved by the ethical review board of the University Medical Center Utrecht (number 06/193), data were analyzed anonymously, informed consent was waived.

### Study population

**Figure 1:**
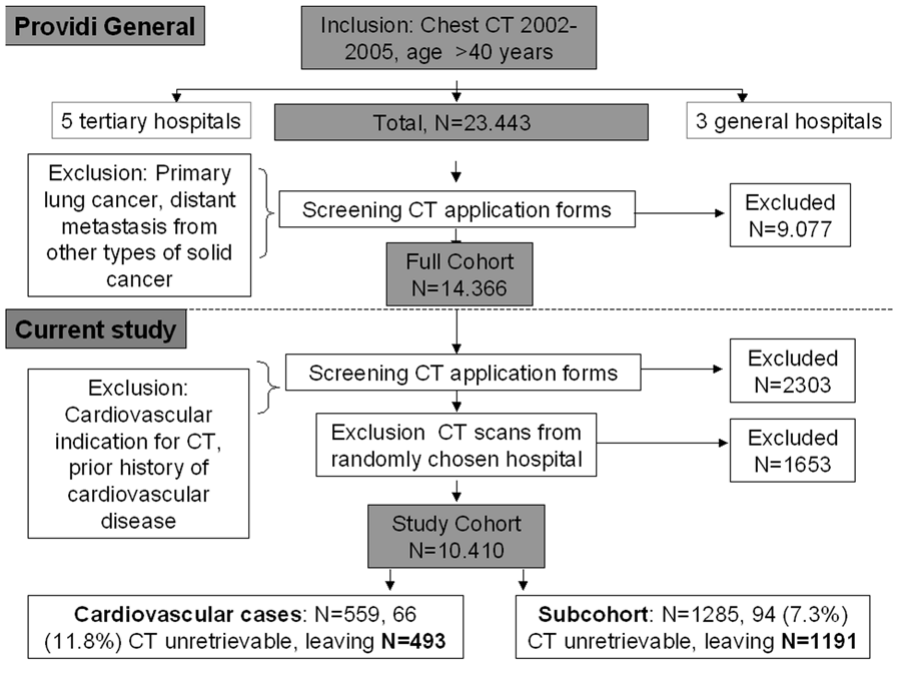
PROVIDI study design and flowchart of the study population.

The rationale and design of the PROVIDI (PROgnostic Value of unrequested Information in Diagnostic Imaging) study has been described in detail and is presented in Figure 1 [4]. Briefly, we identified 23443 subjects 40 years of age and older who underwent chest CT in eight hospitals. Subjects who had lung cancer or metastasis were excluded (N = 9077) because of the poor prognosis. Patients who were suspected of or whom were known to have the outcome of interest (cardiovascular disease) on the pre-CT referral forms were excluded (N = 2303). Finally, for future external validation studies, 1653 subjects from one centre were excluded. For the present study the cohort consisted therefore of chest CT examinations from 10410 individuals. Cases consisted of those subjects who experienced a cardiovascular event hospitalization or died from cardiovascular disease during follow-up. Cardiovascular diseases included cerebrovascular disease, coronary heart disease, peripheral artery disease, heart failure, aortic aneurysm and non-rheumatic heart disease.

### Design

A case-cohort design (Figure 1) [23] was used to limit the number of CT examinations that had to be scored by the observers, while still enabling absolute risk estimations. The random sample of the cohort (subcohort) included 1285 individuals and 94 (7.3%) of these were excluded because the CT examination could not be retrieved, leaving a subcohort of 1191. The cardiovascular endpoints were recorded during an average follow-up time of 17.8 months. The endpoints were obtained through linkage with the National Death Registry and the National Registry of Hospital Discharge Diagnoses [24]–[27]. In cases with multiple valid endpoints, cause of death prevailed over hospital admissions; otherwise the first hospital discharge diagnosis to occur was used.

### Chest CT scoring

All chest CT examinations were obtained using multi-detector CT (2–64 detector rows) of different vendors according to the prevailing routine clinical protocols of the participating hospitals. All CT examinations (all images from all CT data acquisitions) were scored by one of two observers for abnormalities in the mediastinum, lungs and pleura (Table 1) that were considered to be potentially related to inflammation and/or obstructive pulmonary function. For emphysema the scoring system from the National Emphysema Treatment Trial [28] was used, other pulmonary abnormalities were scored as absent or present in 5 lobes. Abnormalities were defined according to the Fleischner Society criteria [29]. A random set of 150 examinations were scored by both observers independently to evaluate the inter-observer agreement. For bronchiectasis, airway wall thickening, ground glass and pleural effusion score 1 was defined as mild, score 2 as moderate and score 3 and higher as severe disease. For emphysema score 1–3 was defined as mild, score 3–6 as moderate and score 7 and higher as severe disease. For lymph-adenopathy a short axis diameter between 6 and 10-mm was defined as mild and greater than 10-mm as moderate disease. In routine clinical practice lymph nodes <10-mm are seen as normal, but we argued that these small lymph nodes may still be predictive for cardiovascular disease.

**Table 1:** CT scoring system for pleural, lung and mediastinal findings.

score	0	1	2	3	4
Emphysema for five lobes (score 0–20)	absent	1–25%	26–50%	51–75%	>76%
Bronchiectasis for five lobes (score 0–5)	absent	present			
Airway wall thickening for five lobes (score 0–5)	absent	present			
Ground glass for five lobes (score 0–5)	absent	present			
Consolidation for five lobes (score 0–5)	absent	present			
Pleural effusion (score 0–6) *	absent	<3 cm	3–6 cm	>6 cm	
Pleural plaques (score 0–2)	absent	unilateral	Bilateral		
Mediastinal lymph node (if ≥5 mm)	absent	… mm			

*For pleural effusion, the thickness of the fluid layer was measured on axial images. For bilateral effusions the most severe side was scored.

### Data analysis

Observer agreement was calculated using a weighted Kappa statistic or an intra-class correlation coefficient (icc). Crude and age and sex adjusted hazard ratios for cardiovascular events were estimated by using Cox proportional-hazards regression. Interaction with CT indication and slice thickness was tested for CT findings that were significantly associated with any cardiovascular event. Annualized event rates were calculated for each CT abnormality [30]. Missing values for CT scores (all <4%) were imputed using regression methods in SPSS (SPSS 14.0, Chicago, Illinois).

## Results

### Subjects

During follow-up we recorded 559 cases of incident cardiovascular disease, 66 (11.8 %) of these were excluded because the CT examination could not be retrieved. Further details of the subcohort and cases are presented in Table 2.

**Table 2:** Characteristics of sub-cohort and cases.

Variable		Sub-cohort (N = 1191)	Cardiovascular disease cases (N = 493)
**Age**, mean age in yrs (range)		61.4 (40–96)	67.9 (40–96)
**Gender**, male sex (%)		58	65
**CT indication** (%)	Pulmonary disease	37	47
	Hematologicalmalignancy	11	5
	Mediastinal disease	11	9
	Ruled-out lung cancer	24	21
	Pulmonary embolism	6	8
	Other	11	10
**Type of medical centre**, tertiary (%)		77	77
**Use of contrast agent** (%)		68	67
**Section thickness** (%)	1–3 mm	43	41
	4–6 mm	39	37
	>6 mm	18	22
**Emphysema** (%)		30	38
mild/moderate/severe (%)		13/10/8	16/11/11
**Bronchiectasis** (%)		28	28
mild/moderate/severe (%)		11/8/9	11/8/9
**Airway wall thickening** (%)		29	46
mild/moderate/severe (%)		9/11/9	11/17/17
**Ground glass** (%)		26	36
mild/moderate/severe (%)		12/7/7	13/10/14
**Consolidation** (%)		28	38
mild/moderate/severe (%)		16/8/4	20/13/5
**Pleural effusion** (%)		14	26
mild/moderate/severe (%)		5/6/4	5/11/9
**Pleural plaques** (%)		15	19
unilateral / bilateral (%)		8/7	8/11
**Lymphadenopathy** (%)		80	85
6–10 mm / >10 mm (%)		60/20	56/29

### CT findings

Lung abnormalities were found to be common with an overall prevalence between 26% and 30% for the various abnormalities in the subcohort. Lymph-nodes >10-mm were seen in 20% whilst pleural abnormalities were less common (14 and 15% for effusion and plaques respectively). There was minimal overlap between these CT findings; it was more common to have either airway wall thickening (42%) or ground glass (36%) than having both (21%). This pattern was similar for the subcohort and the cases and for the other CT findings. Observer agreement was good for pleural effusion (kappa 0.89) and emphysema (icc 0.80), moderate for consolidations (kappa 0.45), lymphadenopathy (kappa 0.54) and plaques (kappa 0.42) and fair for airway wall thickening (kappa 0.33), bronchiectasis (kappa 0.31) and ground glass (kappa 0.38).

### Prediction of cardiovascular events

Several CT findings predicted cardiovascular events. Compared to subjects without the given CT abnormality the hazard for any cardiovascular event was significantly increased in patients with airway wall thickening in more than 1 lobe, ground glass in more than 2 lobes, any consolidation, more than a small unilateral pleural effusion and mediastinal lymphadenopathy >10-mm, even when age and sex were taken into account. The strongest adjusted hazard ratio was for moderate pleural effusion (adjusted hazard ratio 2.77). This corresponds to an annual event rate of 10%, while the annual event rate was 2.3% for the group with no effusion.

On the other hand several other CT items were not predictive for cardiovascular events. The hazard ratio's confidence interval for emphysema, bronchiectasis and pleural plaques covered the value of 1.0 corresponding to no increase in hazard compared to the group without the abnormality.

**Figure 2:**
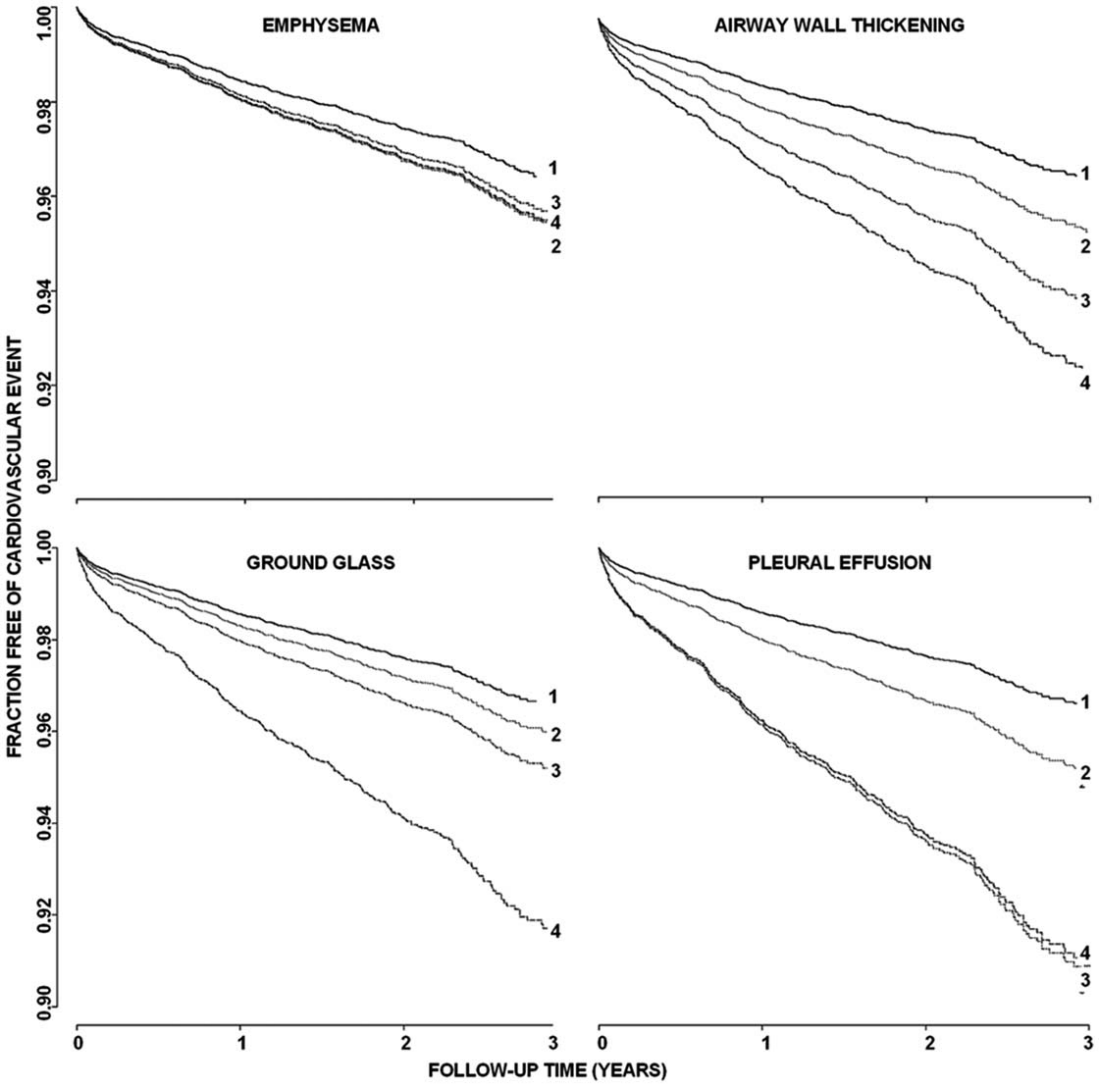
Kaplan-Meier curves for unrequested chest CT findings and the occurrence of future cardiovascular events. Group 1 represents abnormality absent, group 2, 3 and 4 represent mild, moderate and severe abnormalities, respectively. These are simulated Kaplan Meier curves for (age and sex adjusted) 60 year old women.

No significant interaction was found between slice thickness and CT indication and the hazards ratios for airway wall thickening, ground glass, consolidation, pleural effusion and lymph-adenopathy. Such interaction was tested in the age and sex adjusted models. The hazard ratios are presented in Table 3 and annualized mortality and age and sex adjusted Kaplan Meier curves are presented in Table 4 and Figure 2.

**Table 3:** Hazard ratio for any cardiovascular event for CT findings in the lung, pleura and mediastinum.

CT severity	Mild		Moderate		Severe	
Model variables	CT only (crude)	CT, age and sex	CT only (crude)	CT, age and sex	CT only (crude)	CT, age and sex
Emphysema	1.44 (1.06–1.95)	1.27 (0.91–1.76)	1.31 (0.92–1.85)	1.20 (0.83–1.74)	1.49 (1.04–2.16)	1.25 (0.85–1.85)
Bronchiectasis	1.33 (0.97–1.83)	1.00 (0.71–1.40)	1.22 (0.85–1.77)	1.14 (0.76–1.69)	1.39 (0.98–1.96)	1.42 (0.98–2.06)
Airway wall thickening	1.57 (1.10–2.23)	1.33 (0.92–1.93)	2.08 (1.52–2.85)	1.80 (1.29–2.51)	2.74 (1.98–3.80)	2.26 (1.59–3.22)
Ground glass	1.33 (0.96–1.84)	1.18 (0.84–1.67)	1.54 (1.05–2.27)	1.42 (0.94–2.13)	2.62 (1.85–3.73)	2.50 (1.72–3.62)
Consolidation	1.62 (1.22–2.13)	1.39 (1.03–1.87)	1.98 (1.39–2.81)	1.75 (1.21–2.54)	1.79 (1.06–3.04)	1.97 (1.12–3.47)
Pleural effusion	1.47 (0.91–2.38)	1.42 (0.86–2.35)	3.62 (2.41–5.43)	2.77 (1.81–4.25)	3.32 (2.13–5.17)	2.71 (1.72–4.27)
Pleural plaques	1.07 (0.73–1.57)	0.84 (0.55–1.27)	1.38 (0.97–1.98)	1.06 (0.73–1.57)	N.A.	N.A.
Lymph-adenopathy	1.26 (0.94–1.68)	1.12 (0.83–1.52))	2.17 (1.56–3.02)	1.85 (1.30–2.62)	N.A.	N.A.

Data given are hazard ratio's (95% confidence interval). The hazard ratio for CT severity absent is 1.0. N.A.  =  not applicable.

**Table 4:** Annualised event rates for any cardiovascular event.

CT severity	Normal	Mild	Moderate	Severe
Emphysema (%/year)	2.4	3.6	3.3	3.6
Bronchiectasis (%/year)	2.5	3.3	3.1	3.3
Airway wall thickening (%/year)	2.1	3.2	4.3	5.5
Ground glass (%/year)	2.3	3.2	3.5	6.0
Consolidation (%/year)	2.3	3.9	4.4	3.7
Pleural effusion (%/year)	2.3	3.2	10.2	7.5
Pleural plaques (%/year)	2.7	2.8	3.5	N.A.
Lymphadenopathy (%/year)	2.1	2.5	4.4	N.A.

N.A.  =  not applicable.

## Discussion

In a cohort of patients who underwent routine chest CT examinations we showed that unrequested findings in the lungs, mediastinum and pleura are common and that airway wall thickening, ground glass opacities, areas of consolidation, pleural effusion and mediastinal lymph nodes predict cardiovascular events. On the other hand emphysema, bronchiectasis and pleural plaques did not predict cardiovascular events.

In the present study we propose a novel strategy which is the reason why it is not possible to calculate incremental value over known cardiovascular risk factors. We will now illustrate our design to explain why calculating incremental value is not possible and conform clinical practice from the perspective of a radiologist. We see the clinical scenario as follows: A patient is submitted for a chest CT. The radiologist, parallel to the diagnostic procedure, calculates the cardiovascular risk based on CT findings, age and sex. In clinical routine the radiologist does not have access to cardiovascular risk factors and these are even unknown for a large proportion of patients referred for chest CT. If based on the CT findings the cardiovascular risk reaches a certain level this is reported to the referring physician, who can next evaluate conventional risk factors (as long as there is no evidence that CT findings can be treated). The patient is subsequently managed and treated based on current guidelines. The patient may benefit from this CT based strategy as cardiovascular death often occur sudden in patients unknown with cardiovascular disease and even patients known with cardiovascular disease can be undertreated.

This type of prediction research based on routine CT imaging findings has gained interest recently [2]–[4], [31] and opens a wide field of imaging research with new hypotheses to be tested [2]. The ultimate goal is to determine whether routine CT findings can detect and successfully treat patients at high risk for treatable disease [4]. Currently imaging findings are often summarized in detail in the radiologists' written report while the meaning to referring physicians can be unknown. This potentially leads to unneeded further work-up while on the other hand potentially useful findings are ignored. It would be more meaningful to report only relevant unrequested findings for which the prognostic value is known and incorporate absolute risk estimates in the report, especially for outcomes of which the prognosis can be altered. Pragmatically, these risk estimation should be based on information that is readily available to radiologists such as age, sex, probably some medical information (previous malignancy) and imaging findings. The referring physician can then assess, based on the by the radiologist reported risk, whether this reveals hidden disease or whether the patient was already known with increased cardiovascular risk. Our hypothesis that unrequested findings in the lung, pleura and mediastinum might harbor possible prognostic factors was borne out by the observation that several chest diseases are associated with cardiovascular events. Our study may serve as a starting point for including findings in the lungs, mediastinum and pleura in a comprehensive prognostic model that includes known prognostic cardiovascular disease factors such as coronary calcifications. Although our hypothesis was based on potentially causal associations between abnormalities in the lungs [5], [6], [8]–[10], [12], [32]–[34], mediastinum and pleura [15]–[17], our study cannot establish causality between the morphological findings and future cardiovascular events. Possibly, the significant findings in our study are simply a consequence of (subclinical) heart failure [35], [36]. Nevertheless we would like to stress that for the predictive purposes of this study, merely the strength of the predictor and the absolute risk that is associated with that predictor are of interest while the underlying mechanisms are of secondary interest.

Our study has limitations. First, we cannot be certain that there was no history of cardiovascular disease or known cardiovascular risk profile amongst our patients, although we minimized this limitation by excluding all CT examinations obtained for a cardiovascular indication. Also cardiovascular events still occur often sudden and unexpected [20]. Because of these 2 arguments, we believe that although misclassification is an issue this does not invalidate the study. Second, the events are registered on a national basis and there may be errors leading to misclassification on the outcome. Given the strength of the CT predictors in our study, some misclassification would not have seriously altered our conclusion. Third, we cannot be certain that our CT findings were truly incidental or unrequested in all subjects.

In conclusion, unrequested imaging findings in the lungs, mediastinum and pleura are common on diagnostic chest CT. Some CT examinations exhibit prognostic information while others do not. Improving understanding of this information can lead to more intelligent imaging, chest CT reporting, prevention of unnecessary further testing and costs for those findings without prognostic relevance and efficient use of prognostic findings to prevent diseases. CT findings in lungs, mediastinum and pleura may facilitate the detection of subjects at risk for cardiovascular events, especially the detection of airway wall thickening in more than 1 lobe, ground glass in more than 2 lobes, any area of consolidation, more than a small unilateral pleural effusion and mediastinal lymphadenopathy >10-mm. Logical further studies would be the development of a prognostic model that includes vascular calcifications and the evaluation of such models regarding impact on patient management and outcome is needed before widespread application.
